# 

**Published:** 1886-03

**Authors:** 


### Bristol Medico-Chirurgical Journal.

MARCH, 1886.

### CONTENTS.

Page.

ORIGINAL PAPERS :—

Removal of the Uterine Appendages. By J. GREIG SMITH ... ... 1

Relation between Facial Neuralgia and Dental Irritation. By J. M. ACKLAND, M.R.C.S., L.D.S. Eng. ... ... ... ... ... ... ... 28

CLINICAL RECORDS :—

A Case of Dislocation of the Ulna Backwards and the Radius Forwards. By R. J. H. SCOTT, M.R.C.S. Eng. ... ... ... ... 36

Calculus on Foreign Body in Bladder; Perforation of Bladder. No Symptoms. By H. BENHAM, M.D., and J. GREIG SMITH ... 38

Tonic Contraction of the Lower Extremities, or Tetany. By W. H. WEBB, M.R.C.S., L.S.A. ... ... ... ... ... ... ... ... 42

PERISCOPE OF MEDICAL AND SURGICAL PROGRESS :—

Notes on Medical Therapeutics. By ARTHUR B. PROWSE, M.D. Lond., F.R.C.S. Eng. ... ... ... ... ... ... ... ... ... ... ... ... 45

Excoriationes Narium. By BARCLAY J. BARON, M.B. ... ... ... 52

Some Recent Additions to the Dietary of the Sick. By R. SHINGLETON SMITH, M.D. Lond., F.R.C.P. ... ... ... ... ... ... ... ... 55

REVIEWS AND NOTICES OF BOOKS ... ... ... ... ... ... ... ... ... 60

SCRAPS ... ... ... ... ... ... ... ... ... ... ... ... ... ... 71

